# Effective deep learning for oral exfoliative cytology classification

**DOI:** 10.1038/s41598-022-17602-4

**Published:** 2022-08-02

**Authors:** Shintaro Sukegawa, Futa Tanaka, Keisuke Nakano, Takeshi Hara, Kazumasa Yoshii, Katsusuke Yamashita, Sawako Ono, Kiyofumi Takabatake, Hotaka Kawai, Hitoshi Nagatsuka, Yoshihiko Furuki

**Affiliations:** 1 Department of Oral and Maxillofacial Surgery, Kagawa Prefectural Central Hospital, 1-2-1, Asahi-machi, Takamatsu, Kagawa 760-8557 Japan; 2 Department of Oral Pathology and Medicine, Graduate School of Medicine, Dentistry and Pharmaceutical Sciences, Okayama University, Okayama, 700-8558 Japan; 3 Department of Electrical, Electronic and Computer Engineering, Faculty of Engineering, Gifu University, 1-1 Yanagido, Gifu, Gifu 501-1193 Japan; 4 Center for Healthcare Information Technology, Tokai National Higher Education and Research System, 1-1 Yanagido, Gifu, Gifu 501-1193 Japan; 5 Polytechnic Center Kagawa, 2-4-3, Hananomiya-cho, Takamatsu, Kagawa Japan; 6 Department of Pathology, Kagawa Prefectural Central Hospital, 1-2-1, Asahi-machi, Takamatsu, Kagawa 760-8557 Japan

**Keywords:** Pathology, Oral cancer detection

## Abstract

The use of sharpness aware minimization (SAM) as an optimizer that achieves high performance for convolutional neural networks (CNNs) is attracting attention in various fields of deep learning. We used deep learning to perform classification diagnosis in oral exfoliative cytology and to analyze performance, using SAM as an optimization algorithm to improve classification accuracy. The whole image of the oral exfoliation cytology slide was cut into tiles and labeled by an oral pathologist. CNN was VGG16, and stochastic gradient descent (SGD) and SAM were used as optimizers. Each was analyzed with and without a learning rate scheduler in 300 epochs. The performance metrics used were accuracy, precision, recall, specificity, F1 score, AUC, and statistical and effect size. All optimizers performed better with the rate scheduler. In particular, the SAM effect size had high accuracy (11.2) and AUC (11.0). SAM had the best performance of all models with a learning rate scheduler. (AUC = 0.9328) SAM tended to suppress overfitting compared to SGD. In oral exfoliation cytology classification, CNNs using SAM rate scheduler showed the highest classification performance. These results suggest that SAM can play an important role in primary screening of the oral cytological diagnostic environment.

## Introduction

Oral cancer is a life-threatening malignant tumor of the head and neck region, with an estimated 350,000 new cases and more than 170,000 deaths worldwide annually^1^. Most of the histological types of oral cancer are squamous cell carcinomas. Treating oral cancer when it has already advanced in stage has been reported to have a significant impact on the patient's post-treatment quality of life and is associated with a reduced possibility of complete recovery^2–4^, which make early detection and treatment of paramount importance.

Histological diagnosis is required when malignancy or dysplasia of the oral cavity is suspected. However, biopsy is invasive because it involves surgical resection. Therefore, oral exfoliative cytology, a minimally invasive examination modality, is attractive and effective for initial diagnosis and screening. The purpose of oral cytology is primary screening to determine whether to make a histopathological diagnosis to obtain a definitive diagnosis and to screen out whether malignancy or dysplasia is suspected^5^. The liquid-based cytology (LBC) method, in which cells are dispersed in a fixative solution and a thin layer of cells is generated on a slide, not only has the advantage of less chair-side work in clinical practice but also of standardizing the diagnosis because the prepared specimen is a thin-layered smear with few overlapping cells^6^. The development of the LBC method provides accurate and standardized diagnoses and has made oral cytology more accessible.

The clinical flow of cytological diagnosis consists of rough screening by cytopathologists and definitive diagnosis by pathologists. First, cytological technicians or cytopathologists manually mark a key area of diagnosis using an ink marker. The marked area is then subjected to an appropriate secondary review by a pathologist^7^. Unfortunately, manual testing to detect abnormal areas on oral cytopathological slides is a very tedious process for professional cytologists. Moreover, a lot of training is required to accurately diagnose abnormal cells manually under a microscope. Therefore, computer-aided technology is a boon for efficient diagnosis.

In recent years, deep learning has been developed and applied in various fields. The convolutional neural network (CNN), which originated from the neocognitron proposed by Fukushima^8^, is a neural network in which layers are connected by local coupling of common weights; it has brought about a revolutionary change in the field of image recognition. Deep learning using CNN has had a great effect on the classification of medical images^9,10^. The development of various deep learning CNN models^11,12^ and various optimization algorithms to improve the classification accuracy is rapidly progressing. There are various optimization algorithms, and in recent years, Sharpness Aware Minimization (SAM)^13^ has been reported as an effective learning method for CNNs. SAM is an optimization algorithm published by Google Research. Until now, the parameters were learned so that loss was minimized, but SAM is a new method for updating the parameters in consideration of minimum loss and the flatness of the surroundings.

Therefore, we hypothesized that using SAM as an optimization algorithm would improve the accuracy of the classifier. The purpose of this study was to perform two classifications of oral exfoliative cytology using deep learning and to analyze the performance using SAM as an optimization algorithm to improve classification accuracy.

## Results

### Searching for the optimal ρ in SAM

**Figure 1:**
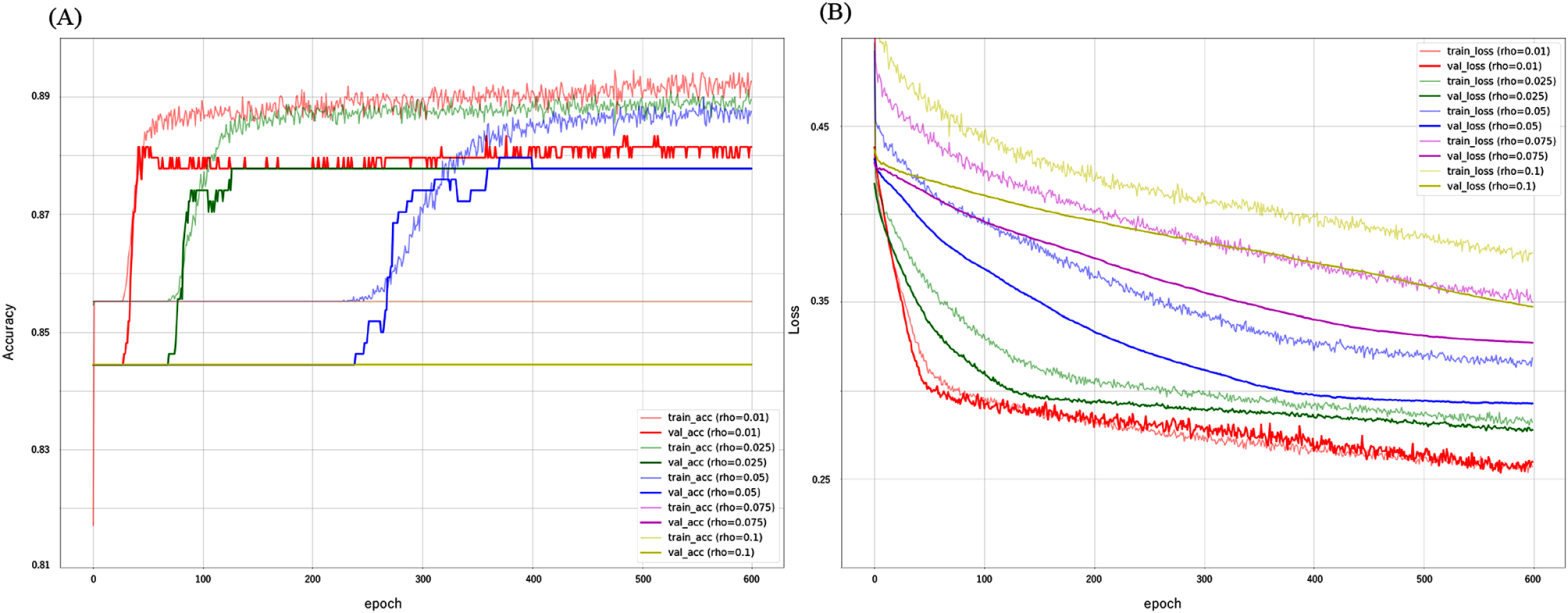
Learning curve by grid search for SAM ρ determination. (**A**) accuracy score (**B**) loss scoreIn Epoch300, the convergence was good when ρ was 0.01 or 0.025. In the comparison of ρ = 0.025 and 0.01 in Loss, 0.025 was more stable.

The results of the grid search for the optimal ρ search when using SAM as an optimizer are shown in the learning curve (Fig. 1). In general, it was shown that the larger ρ, the more epoch is required for convergence. The results of the grid search for the optimal ρ search when using SAM as an optimizer are shown in learning curves. In Epoch300, the convergence was good when ρ was 0.01 or 0.025. Overfitting occurs at ρ = 0.1. In the comparison of ρ = 0.025 and 0.01 in Loss, 0.025 was more stable.

Based on this result, ρ = 0.025 was adopted in this study to compare the performance of deep learning at 300 epochs.

### Comparison of learning curves between optimizer SAM and SGD with and without a learning rate scheduler

**Figure 2:**
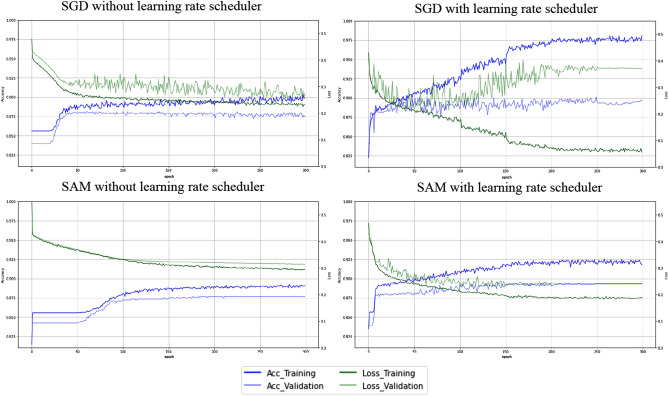
Learning curve in each CNN model.

Figure 2 shows the learning curve for each deep learning model. Interestingly, as the learning progressed, the dissociation in the training and validation data in accuracy and loss was smaller in SAM than in stochastic gradient descent (SGD). In other words, SGD showed overfitting with increasing epochs. On the other hand, even with increasing epochs, SAMs tended to be less likely to show overfitting. We also found that the time to learning was shortened by adding a learning rate scheduler.

### Comparison of optimizer SAM and SGD with and without a learning rate scheduler

Table 1 shows the results of the performance metrics with and without the learning rate scheduler in the SGD and SAM optimizers. In SGD, the introduction of learning rate scheduling improved all performance metrics except precision. In addition, in SAM, the introduction of learning rate scheduling improved all performance. Of all the models, the one with the highest AUC was the one that introduced the learning rate into the SAM. (AUC = 0.9328) (Supplementary Fig. S1).

**Table 1:** Comparison of optimizer SAM and SGD with and without a learning rate scheduler.

Optimizer	Learning rate	Accuracy	Precision	Recall	F1 score	AUC
		SD	SD	SD	SD	SD
		95% CI	95% CI	95% CI	95% CI	95% CI
**SGD**						
	w/o scheduler	0.8879	0.8518	0.6501	0.6937	0.9020
		0.0016	0.0099	0.0089	0.0095	0.0075
		0.8873–0.8885	0.8482–0.8553	0.6469–0.6533	0.6903–0.6971	0.8993–0.9047
	With scheduler	0.8970	0.8049	0.7581	0.7780	0.9098
		0.0026	0.0066	0.0064	0.0057	0.0030
		0.8961–0.8979	0.8026–0.8073	0.7558–0.7604	0.7759–0.7800	0.9087–0.9108
**SAM**						
	w/o scheduler	0.8870	0.8529	0.6438	0.6872	0.8766
		0.0004	0.0024	0.0017	0.0019	0.0069
		0.8868–0.8871	0.8521–0.8538	0.6432–0.6444	0.6875–0.6879	0.8741–0.8791
	With scheduler	0.9016	0.8534	0.7139	0.7578	0.9328
		0.0018	0.0072	0.0124	0.0098	0.0016
		0.9010–0.9023	0.8509–0.8560	0.7094–0.7183	0.7543–0.7614	0.9322–0.9334

SD, standard deviation; 95% CI, 95% confidence interval; AUC, Area under the ROC curve.

### Comparison of optimizers SAM and SGD with and without a learning rate scheduler

For each performance metric, we performed a statistical evaluation for each model difference in Table 2. The introduction of the learning rate scheduler showed a statistically significant difference in *P*-values below 0.05, except for precision in SAM. Especially in the case of SAM, by adding a learning rate scheduler, very large effects in accuracy and AUC were obtained (accuracy:11.226, AUC: 10.997). In addition, statistically significant differences were found in all of the statistical comparisons of SGD and SAM by *P*-value with the learning rate scheduler. In the effect size comparison, the AUC was 9.529, and the difference in the optimizer under the same deep learning conditions showed a very large effect size equivalent to "Huge" by SAM.

**Table 2:** Statistical evaluation of optimizers SAM and SGD with and without a learning rate scheduler.

Performance metrics	Model B	Model A	A–B	*P* value	Effect size
**SGD**					
Accuracy	w/o LRS	With LRS	0.0091	< .0001	4.143
Precision	w/o LRS	With LRS	− 0.0468	< .0001	5.463
Recall	w/o LRS	With LRS	0.1080	< .0001	13.721
F1 score	w/o LRS	With LRS	0.0843	< .0001	10.609
AUC	w/o LRS	With LRS	0.0078	< .0001	1.348
**SAM**					
Accuracy	w/o LRS	With LRS	0.0147	< .0001	11.226
Precision	w/o LRS	With LRS	0.0005	0.733	0.090
Recall	w/o LRS	With LRS	0.0701	< .0001	7.832
F1 score	w/o LRS	With LRS	0.0706	< .0001	9.894
AUC	w/o LRS	With LRS	0.0562	< .0001	10.997
**SAM versus SDG with scheduler**					
Accuracy	SDG	SAM	0.0046	< .0001	2.047
Precision	SDG	SAM	0.0485	< .0001	6.924
Recall	SDG	SAM	− 0.0442	< .0001	4.432
F1 score	SDG	SAM	− 0.0201	< .0001	2.476
AUC	SDG	SAM	0.0230	< .0001	9.529

AUC, area under the ROC curve; LRS, learning rate scheduler.

### Visualization of each model classification by Grad-CAM and attention heatmap

**Figure 3:**
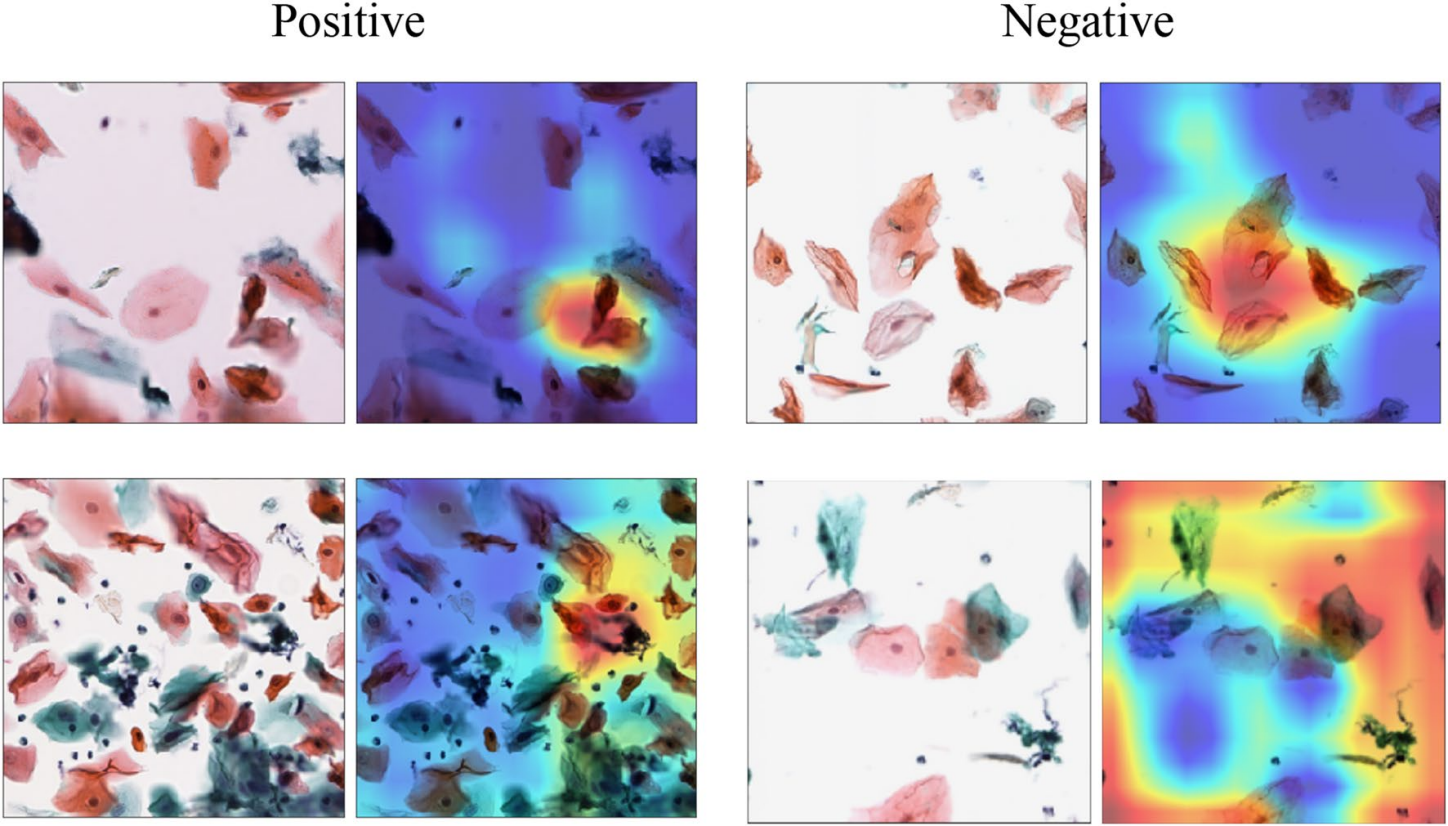
Visualization of regions of interest for CNN classification in oral exfoliative cytopathology classification. In the heat map visualization, the warmer the color, the greater the effect on label classification.

Figure 3 shows an image that visualizes the area of interest for classification decisions in a deep learning model. In the VGG16-based CNN model, Grad-CAM was used to visualize the final layer of the convolutional layer or the feature area of the oral scraping cytopathological classification with a heat map.

In the positive label, it can be seen that we are paying attention to atypical cells with a high nuclear ratio (N/C ratio) in the cytoplasm as a characteristic region and an increased amount of chromatin in the cell nucleus. Among the deep blue-stained cells, we focused on cells with a high N/C ratio and classified them as a positive class. In the negative label, the superficial cells stained in orange and red did not increase the amount of chromatin, and cells with a low N/C ratio were used as the basis for judgment. In addition, the negative label showed that the classification was predicted by focusing on the entire field of view.

## Discussion

The CNN model using SAM introduced by the learning rate scheduler showed the highest classification performance with 90.2% accuracy and AUC 0.93 in a limited number of epochs (epoch 300) and was able to suppress overfitting. The most effective deep learning model for oral exfoliation cytology was the CNN model using SAM as the optimizer and incorporating the learning rate scheduler.

There are no reports on the accuracy of classification using deep learning in oral exfoliative cytopathology. Sunny et al.^14^ reported the application of deep learning in oral cytopathology. Their study investigated the clinical usefulness of the system in combination with CNNs in the classification of atypical cells. This report showed that the use of a CNN-based risk stratification model improved the detection sensitivity of malignant lesions (93%) and high-grade OPML (73%). However, the classification accuracy of CNN was not verified. By contrast, our study is the first to evaluate classification accuracy using deep learning models optimized in oral exfoliative cytopathology.

The diagnosis of oral exfoliative cytopathology is difficult. With the introduction of the LBC method, the issue of cell overlap has decreased^6^ but it still remains. In addition, the number and type of cells are very large compared to cervical cytology, making judgment difficult for instance. A lot of deep-learning research on cervical cytopathology has been undertaken, and the accuracy is very high^15–17^. This is because the state of each cell can be judged. On the other hand, oral exfoliative cytopathology requires experience and skill because it is necessary to judge abnormalities from the entire visual field. This difficulty is an obstacle to the efforts of deep learning for oral cytopathological diagnosis classification.

In this study, we compared the SGD and SAM as optimizers. SAM has been the focus of attention in recent years, updating the state of the art (SoTA) with as many as nine datasets, including ImageNet (88.61%), CIFAR-10 (99.70%), and CIFAR-100 (96.08%)^13^. The introduction of SAM has contributed to a revolutionary improvement in the accuracy of image classification. It has also been suggested that loss flatness plays an important role not only in accuracy but also in generalization performance and robustness. Another advantage of SAM is that it is difficult to overfit. When the number of epochs was increased, the CNN model using an optimizer other than SAM was overfitted, whereas SAM was difficult to overfit, even when the number of epochs was increased^13^. In our study, SGD tended to overfit, while SAM tended to avoid overfitting as the number of epochs increased.

During the early learning stages of deep learning, the network changes rapidly, and the linear scaling rules do not work. It has been reported that this can be mitigated by a less aggressive learning rate usage strategy at the start of training^18^. However, although a low learning rate can be expected to converge stably, there is a problem with learning speed. One solution is to warm up the learning rate gradually from a small value to a large value^19^. This avoids a sudden increase in the learning rate and allows for an optimal convergence at the beginning of training. The SAM used in this study required time to converge. Therefore, sufficient learning could not be performed within a limited number of epochs, and underfitting was possible without the introduction of a learning rate scheduler. On the other hand, if the learning rate remains high, efficient learning will be achieved, but this will prevent the network from handling noisy data. Therefore, lowering the learning rate after some learning helps the network converge to a local minimum and mitigate the effects of vibration^20^. By adopting warm-up and step-decay as the learning rate scheduler in this study, we found that the accuracy was improved in both SGD and SAM optimizers. Therefore, it was suggested that the learning rate scheduler plays an important role in the deep learning of oral exfoliative cytopathology.

In this study, the effect size was calculated in addition to the *P*-value as a method for evaluating the comparison of performance metrics in deep learning. Effect size is an indicator of the effectiveness of an experimental operation and the strength of the association between variables^21^. In the evaluation of the effect of introducing the learning rate scheduler in this study, the *P* values ​​were all 0.05 or less. By considering this and the effect size, it was possible to evaluate the strength of the effect of introducing the scheduler. Oral exfoliative cytopathology has shown that the introduction of a scheduler into SAM is particularly effective. In addition, we believe that the detected effect size will be an important prior study to help calculate sample size in studies in cytopathological classification using deep learning.

In the future, the study of classification models with our CNN may bring a major shift in the diagnostic flow of oral exfoliative cytopathology. In this study, AUC had 93% accuracy for the classification of normal findings and suspected malignancy or dysplasia. If deep learning technology can be applied as a primary screening tool for cytopathological diagnosis, it will contribute to the field of pathology, which is understaffed. In addition, the images divided using OpenSlide are numbered so that the location of the slide can be specified. This presents a shortcut for practical clinical applications. In the future, we look forward to further research so that more robust diagnostic analysis can be performed using data for oral scraping cytopathology performed at multiple centers.

This study had some limitations. First, data collection was in a single facility and was not externally validated. Internal validity can be evaluated by confidence intervals from datasets using cross-validation, but verification using external data will be required in the future. Second, the data bias in this study was large. The number of positive labels was only 881 while that of negative labels was 5113. Therefore, adding or resampling the data should also be considered as an approach to imbalanced data. However, undersampling, the main method of resampling, misses important data^22^. On the other hand, oversampling has a risk of overfitting^22^. Therefore, it will be necessary to consider the addition of specificity obtained from resampling analysis and indicators such as the PR curve that plots the prediction for recall. Third is a need to consider other CNN models, optimizers^23^ and learning rate scheduling^24^. Currently, there are numerous types of optimizers. In addition, there are also many methods for scheduling the learning rate. However, choosing the best CNN model, optimizer and scheduling the learning rate for your dataset is a difficult problem because it is computationally expensive^25^. It will be necessary to search for optimum CNN model selection and best parameter tuning in the future.

## Conclusions

In this study, we explored an effective deep learning model for oral exfoliative cytopathological classification using SGD or SAM as an optimizer, with and without a learning rate scheduler. The CNN model using SAM introduced by the learning rate scheduler showed the highest classification performance in a limited number of epochs and was able to suppress overfitting. These results suggest that SAM can play a very important role in primary screening of the oral cytological diagnostic environment.

## Materials and methods

### Study design

The aim of this study was to analyze the classification performance for oral exfoliative cytology diagnosis using a deep learning model using a supervised learning CNN and to analyze the effect of using SAM as an optimization algorithm.

### Ethics statement

This study was approved by the Kagawa Prefectural Central Hospital Ethics Committee (approval number: 977). This institutional review board reviewed our study, which has a non-interventional retrospective study design and is an analytical study with anonymized data, and waived the need for informed consent. Therefore, written and verbal informed consent was not obtained from the study participants. This study was conducted in accordance with the Declaration of Helsinki and according to the rules approved by the ethics committee.

### Image data preparation

In this study, we used eight glass slides prepared using the LBC method. The breakdown of the eight slides included four cases of tongue cancer, two cases of buccal mucosal cancer, and two cases of tongue leukoplakia. The glass slides were scanned using Aperio AT2 scanners (Leica Biosystems, Buffalo Grove, IL) at 40 × magnification to create a Whole Slide Image (WSI). The WSIs were tiled using OpenSlide (version 3.4.1, University of Pittsburgh, Pittsburgh, Pennsylvania). OpenSlide is a C language library developed by a research group at Carnegie Mellon University.

**Figure 4:**
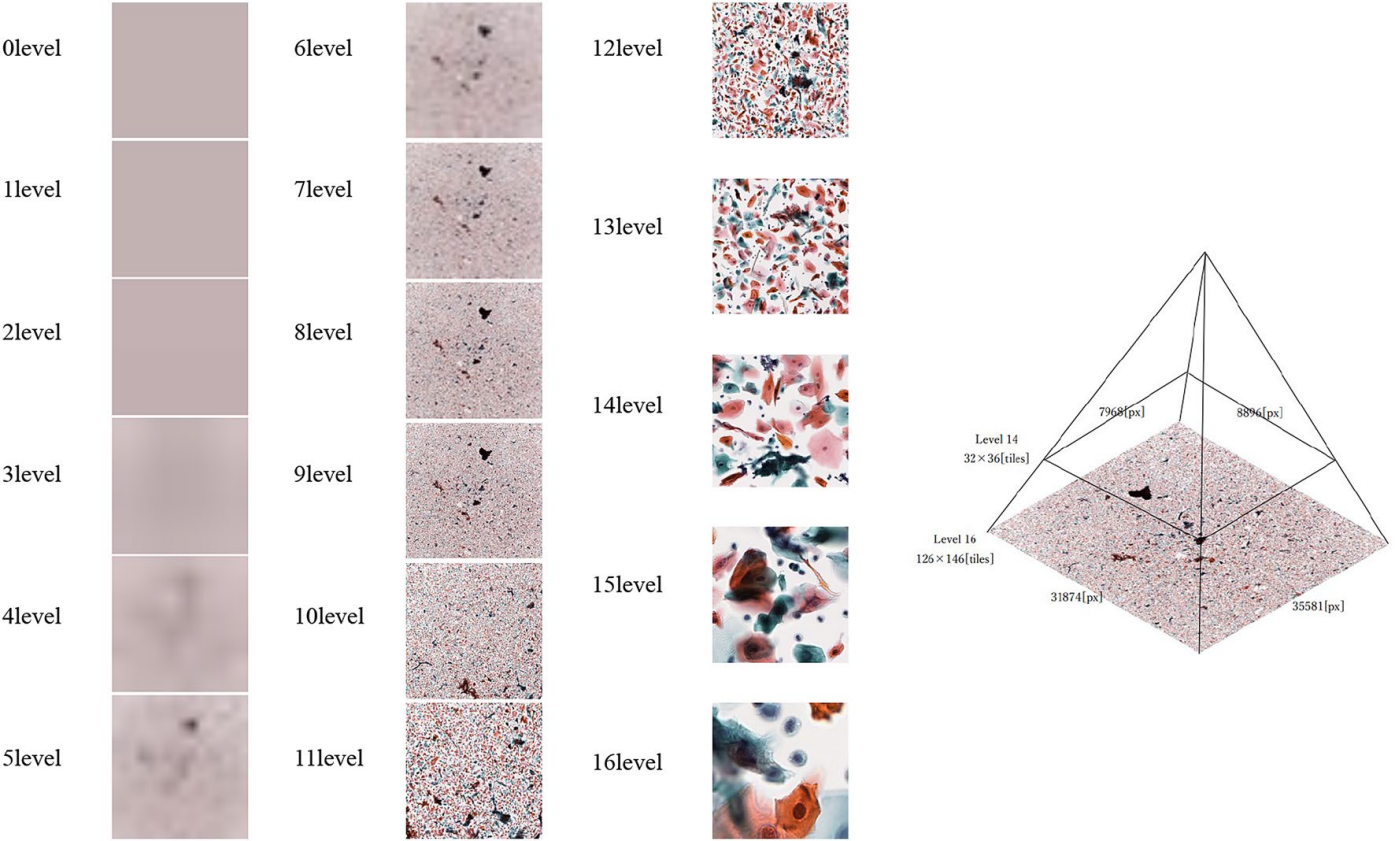
Automatic division of WSI using OpenSlide. Images can be acquired from different depth levels. In this study, images with a depth of 14th level were used.

The WSI was then tiled using the open-source library Openslide^26^ (version 3.4.1, University of Pittsburgh, Pittsburgh, Pennsylvania). OpenSlide is a C language library developed by a research group at Carnegie Mellon University. Because WSI is compatible with each magnification, it is possible to evaluate cytopathology at the optimum magnification, so we divided it into 16 layers at a magnification of 10 to 400 times. The pathologist determined the optimal magnification for diagnosis from these images as the 14th level, and the image was cut out and extracted in tiles. The clipped image was output in a Portable Network Graphics (PNG) format of 256 × 256 pixels (Fig. 4).

### Image data annotation and selection

**Figure 5:**
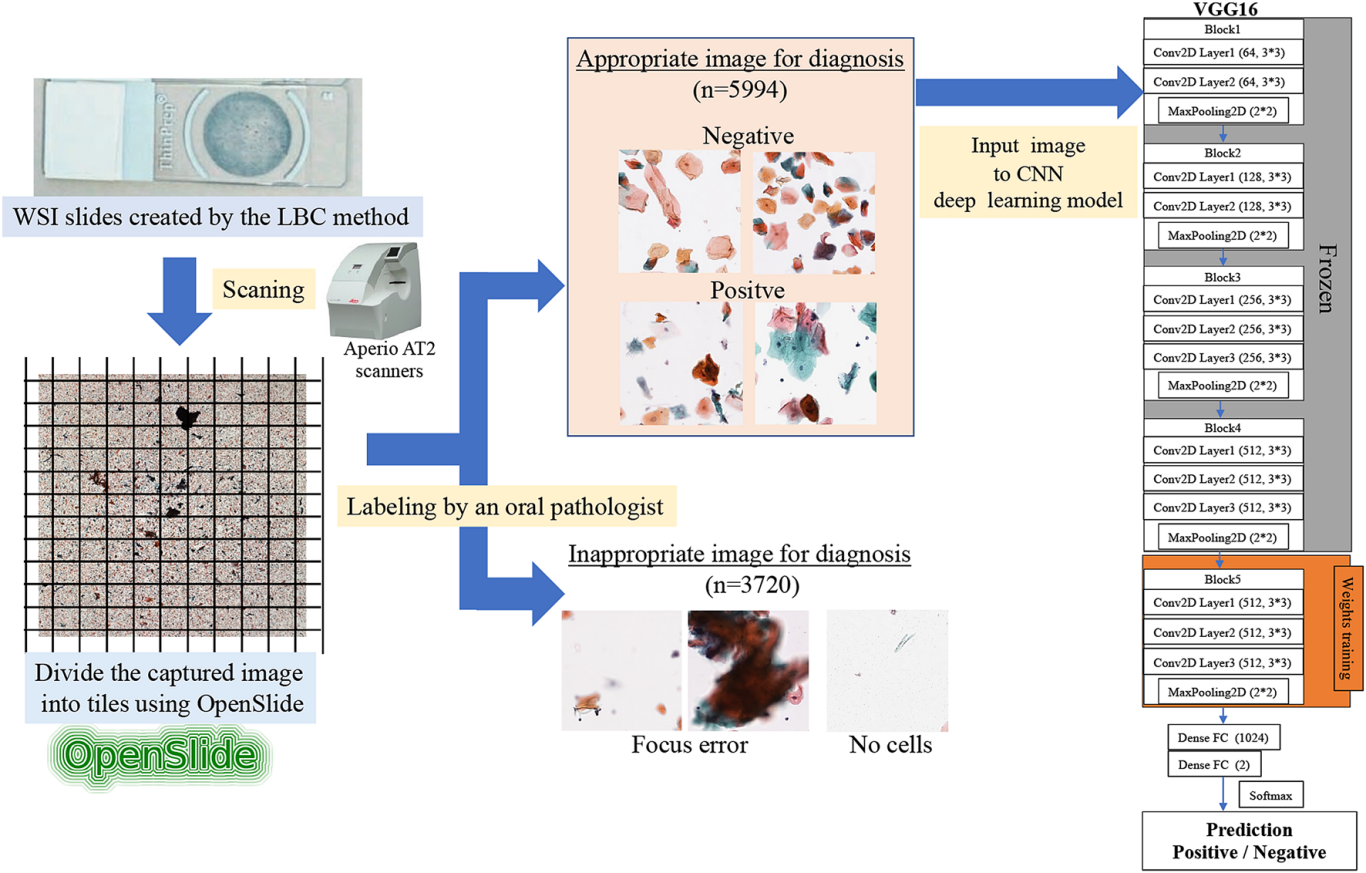
Overall flow of deep-learning classification model research of oral exfoliative cytopathology.

The oral cytology diagnosis from the fragmented images was annotated by two cytopathologists. The images were labelled according to consistency in the diagnosis of the two pathologists and an additional diagnosis of a highly specialized doctor was sought in the case of controversy. Images for which proper diagnosis was not possible due to excessive overlap of cells, poor focus, etc., or images without cells were excluded from this study. Tiles were first classified into five categories based on the Papanicolaou classification. Classes I and II were classified with a negative label, and classes III, IV, and V were classified with a positive label (Table 3). Figure 5 shows the overall flow of this study.

**Table 3:** Label data distribution.

Class	Number of images	Description (From papanicolaou, 1954)
**Negative label**		
ClassI	2210	Absence of atypical or abnormal cells
ClassII	2903	Atypical cytology, but no evidence for malignancy
**Positive label**		
ClassIII	225	Cytology suggestive of, but not conclusive for, malignancy
ClassIV	416	Cytology strongly suggestive of malignancy
ClassV	240	Cytology conclusive for malignancy
Total	5994	
Unclassifiable images	3720

### CNNs model architecture

VGG16^27^ is a representative network of CNNs developed at Oxford University and submitted to the ImageNet Large-Scale Visual Recognition Challenge (ILSVRC) in 2014. VGG16 has a structure in which the "convolution layer/convolution layer/pooling layer" is repeated twice, and the "convolution layer/convolution layer/convolution layer/pooling layer" is repeated three times, followed by three fully connected layers. It was reported that VGG16 is a model that can be expected to further improve robustness in recent year^28^. Therefore, we selected VGG16 as the CNN model in this study.

VGG16 CNN models have adopted fine-tuning using the ImageNet database. The deep learning classification task process was implemented using Keras (version 2.7.0), Tensorflow (version 2.4.0), and Python language (version 3.7.10).

### Data set and model training

The CNN model training was generalized using K-fold cross-validation in the deep learning algorithm. Model validation was evaluated using 4-fold cross-validation to avoid overfitting and bias and to minimize the generalization error. The dataset was divided into four random subsets using stratified sampling, and the same class distribution was maintained for training, validation, and testing across all subsets^29^. Within each fold, the dataset was split into separate training and test datasets in a ratio of 9:1. Additionally, the validation data consisted of 10% of the training data. The model averaged four training iterations to obtain prediction results for the entire dataset, with each iteration retaining a different subset for validation.

For the loss function, the cross-entropy obtained from the following equation was used:

$$ L\left( w \right) = - \mathop \sum \limits_{i = 0} t_{i} \log y_{i} $$

t_i_: true label, y_i_: predicted probability of class i.

In our study, different image data augmentation methods including, rotation, flipping, and shifting, were randomly applied to generate training images. The details are explained in the supplementary materials.

#### Optimization algorithm

Although there are many types of optimizers^30^, in this research, we made a comparison with SAM, representing SGD, which is currently used by many researchers.

##### Stochastic gradient descent (SGD)

In deep learning, learning is advanced so that the error between the correct answer and the prediction becomes small. One commonly utilized algorithms is SGD. SGD updates the parameters using the derivative of the loss function. In addition, by using randomly selected data to update the parameters, it is possible to prevent falling into a local minimum value. As an advanced version of SGD, we selected SGD with momentum, which suppresses vibration by considering the moving average^31^. SGD with momentum is expressed by the following formula:

$$ \Delta w_{t} = \eta \nabla {\text{L}}\left( w \right) + \alpha \Delta w_{t - 1} $$

$$ w_{t} = w_{t - 1} - \Delta w_{t} $$

w_t_: t-th  parameter, η: learning rate.∇L (w): Differentiation with parameters of loss function, α: Momentum.

##### Sharpness aware minimization (SAM)

SAM was used to verify the effective learning method of the CNN^13^. The loss function of SAM is defined by the following algorithm (a): The SAM minimizes equation (b), including this. In addition, ρ is called the neighborhood size, which is a hyper-parameter set during tuning. In SAM, the base optimizer and SAM are used in combination to determine the final parameters using a conventional algorithm. This study was based on the SGD.

$$ \mathop {\min }\limits_{w} L_{S}^{SAM} \left( w \right) + \lambda \left\| w \right\|_{2}^{2} \quad ({\text{a}}) $$

$$ L_{S}^{SAM} \left( w \right) = \mathop {\max }\limits_{\left\| \varepsilon \right\|p \le \rho } L_{s} \left( {w + \varepsilon } \right)\quad ({\text{b}}) $$

S: set of data, w: parameter, λ: L2 regularization coefficient.

Ls: Loss function, ρ: neighborhood size.

In this study, the optimum ρ was examined by performing a grid search from {0.01, 0.02, 0.05, 0.1, 0.2, 0.5}.

### Deep learning procedure

#### Learning rate scheduler

In June 2017, Facebook Inc. proposed a warm-up strategy that gradually increased the learning rate at the start of learning and stabilized learning^19^. Warmup sets the initial learning rate to be smaller than usual and gradually increases it to the normal learning rate, and an efficient learning effect can be expected to result from this approach^32^. Warmup as a learning rate scheduler is shown by the following equation:

$$ {\text{r}}_{{{\text{warmup}}}} = { }10^{{\left( { - {\text{base}} + { }\frac{{{\text{base}}}}{{{\text{interval}}}}{ } \times {\text{epoch}}} \right)}} \times {\text{lr}}_{{{\text{init}}}} $$

On the other hand, learning rate decay is a method used to improve the generalization performance of deep learning, and it is a method to lower the learning rate when learning has progressed to some extent. Learning rate attenuation is known to improve accuracy^18^. In this study, we also examined the effects of warmup and step-decay as a learning rate scheduler shown in Supplementary Fig. S2.

The optimizer performed SGD with momentum and SAM. The learning rate was 0.001 for SGD with momentum. The existence of the learning rate scheduler was verified for each optimizer. The learning rate was 0.001, and the warm-up and step-decay as the learning rate scheduler were performed with the learning rate scheduler as the initial learning rate of 0.01. All models analyzed 300 epochs and 32 mini-batch sizes. This process was repeated 30 times on both models of each optimizer using different random seeds for each CNN model.

### Performance metrics and statistical analysis

All CNN models were evaluated using accuracy, precision, recall, specificity, F1 score, and AUC calculated from the receiver operating characteristic curve (ROC) as performance metrics.

### Visualization of a computer-assisted diagnostic system

It is important to visualize the rationale for image prediction using a CNN. Gradient-weighted class activation mapping (Grad-CAM) targets CNN-based image recognition models^33^. This method gives a judgment basis to the model itself by weighting the gradient with respect to the predicted value. In this study, a heat map was used to emphasize the part that served as the basis for judgment according to its importance. Grad-CAM uses the final convolution layer of the VGG16 model.

### Statistical analysis

Statistical assessments of the classification performance of each CNN model were performed on the data analyzed 30 times because there are no previous studies on deep learning of oral cytology classification. All metrics in this study were analyzed using the JMP Statistical Software Package Version 14.2.0 for Macintosh (SAS Institute Inc., Cary, NC, USA). Differences were considered statistically significant at P values less than 0.05. The normal distribution of continuous variables was evaluated using the Shapiro–Wilk test. The difference in classification performance between each CNN model was calculated for each performance metric using the Wilcoxon test. Effect sizes^34^ were calculated as Hedges' g (unbiased Cohen's d) using the following equation:

$$ {\text{Hedges}}\prime \,{\text{g}} = \frac{{|{\text{M}}_{1} - {\text{M}}_{2} |}}{{\text{s}}} $$

$$ {\text{s}} = \sqrt {\frac{{({\text{n}}_{1} - 1){\text{s}}_{1}^{2} + \left( {{\text{n}}_{2} - 1} \right){\text{s}}_{2}^{2} }}{{{\text{n}}_{1} + {\text{n}}_{2} - 2}}} $$

M1 and M2 are the means for the CNN model (optimizer; SGD or with/without l*earning rate scheduler*) and CNN model (optimizer; SAM or with/without l*earning rate scheduler*), respectively. s1 and s2 are the standard deviations for the CNN model (optimizer; SGD or with/without l*earning rate scheduler*) and CNN model (optimizer; SAM or with/without l*earning rate scheduler*), respectively. n1 and n2 are the numbers for the CNN model (optimizer; SGD or with/without l*earning rate scheduler*) and CNN model (optimizer; SAM or with/without l*earning rate scheduler*), respectively.

Effect sizes were categorized based on the criteria proposed by Cohen and expanded by Sawilowsky^35^: large effect was 2.0 or more, very large effect was 1.0, large effect was 0.8, medium effect was 0.5, small effect was 0.2, and a very small effect was 0.01.

## Supplementary Information

Supplementary Information.

## Data Availability

The data are not publicly available due to privacy. The data presented in this study are available on request from the corresponding author.
