# Supplementary material for: Effective deep learning for oral exfoliative cytology classification
Source: Sci Rep. 2022 Aug 2;12:13281. doi: 10.1038/s41598-022-17602-4 (PMC9346110; doi:10.1038/s41598-022-17602-4)
Supplement: Supplementary file 1 — Supplementary Information. [file 41598_2022_17602_MOESM1_ESM.docx]

Effective deep learning for oral exfoliative cytology classification

Shintaro Sukegawa^1,2,^ *, Futa Tanaka^3^, Keisuke Nakano^2^, Takeshi Hara^3,4^, Kazumasa Yoshii^3^, Katsusuke Yamashita^5^, Sawako Ono^6^, Kiyofumi Takabatake^2^, Hotaka Kawai^2^, Hitoshi Nagatsuka^2^, Yoshihiko Furuki^1^

^1^ Department of Oral and Maxillofacial Surgery, Kagawa Prefectural Central Hospital, 1-2-1, Asahi-machi, Takamatsu, Kagawa 760-8557, Japan

^2^ Department of Oral Pathology and Medicine, Graduate School of Medicine, Dentistry and Pharmaceutical Sciences, Okayama University, Okayama 700-8558, Japan

^3^ Department of Electrical, Electronic and Computer Engineering, Faculty of Engineering, Gifu University, 1-1 Yanagido, Gifu, Gifu 501-1193, Japan

^4^ Center for Healthcare Information Technology, Tokai National Higher Education and Research System, 1-1 Yanagido, Gifu, Gifu 501-1193 Japan

^5^ Polytechnic Center Kagawa, 2-4-3, Hananomiya-cho, Takamatsu, Kagawa

^6^ Department of Pathology, Kagawa Prefectural Central Hospital, 1-2-1, Asahi-machi, Takamatsu, Kagawa 760-8557, Japan

**Appendix**

***Figure S1: Average ROC in each deep learning model using SAM and SGD as an optimizer, and with and without the introduction of a learning rate scheduler***


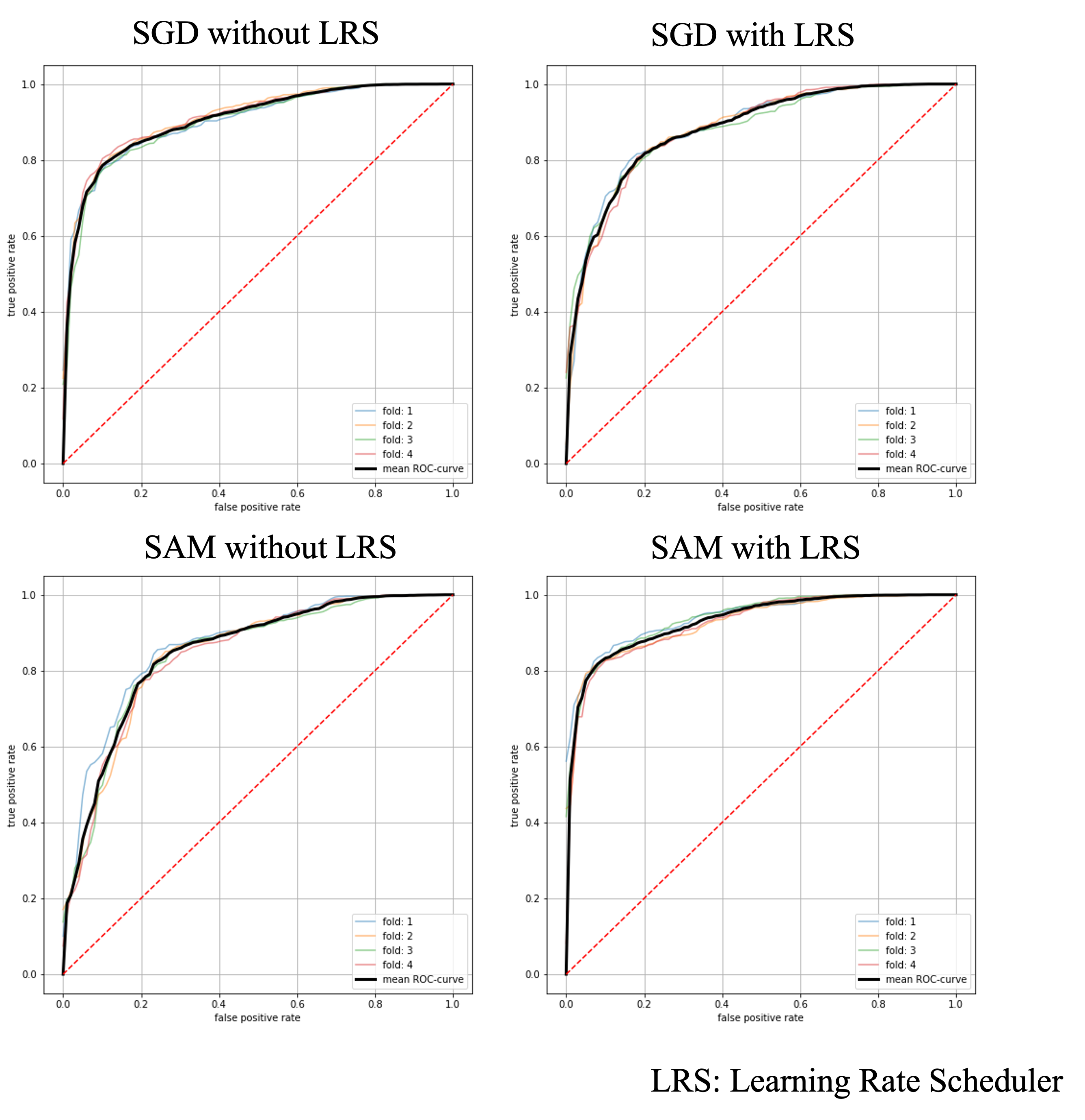


**Figure S2. Changes in learning rate for each number of epochs in scheduler warmup and step-decay as a learning rate in this study.**

***
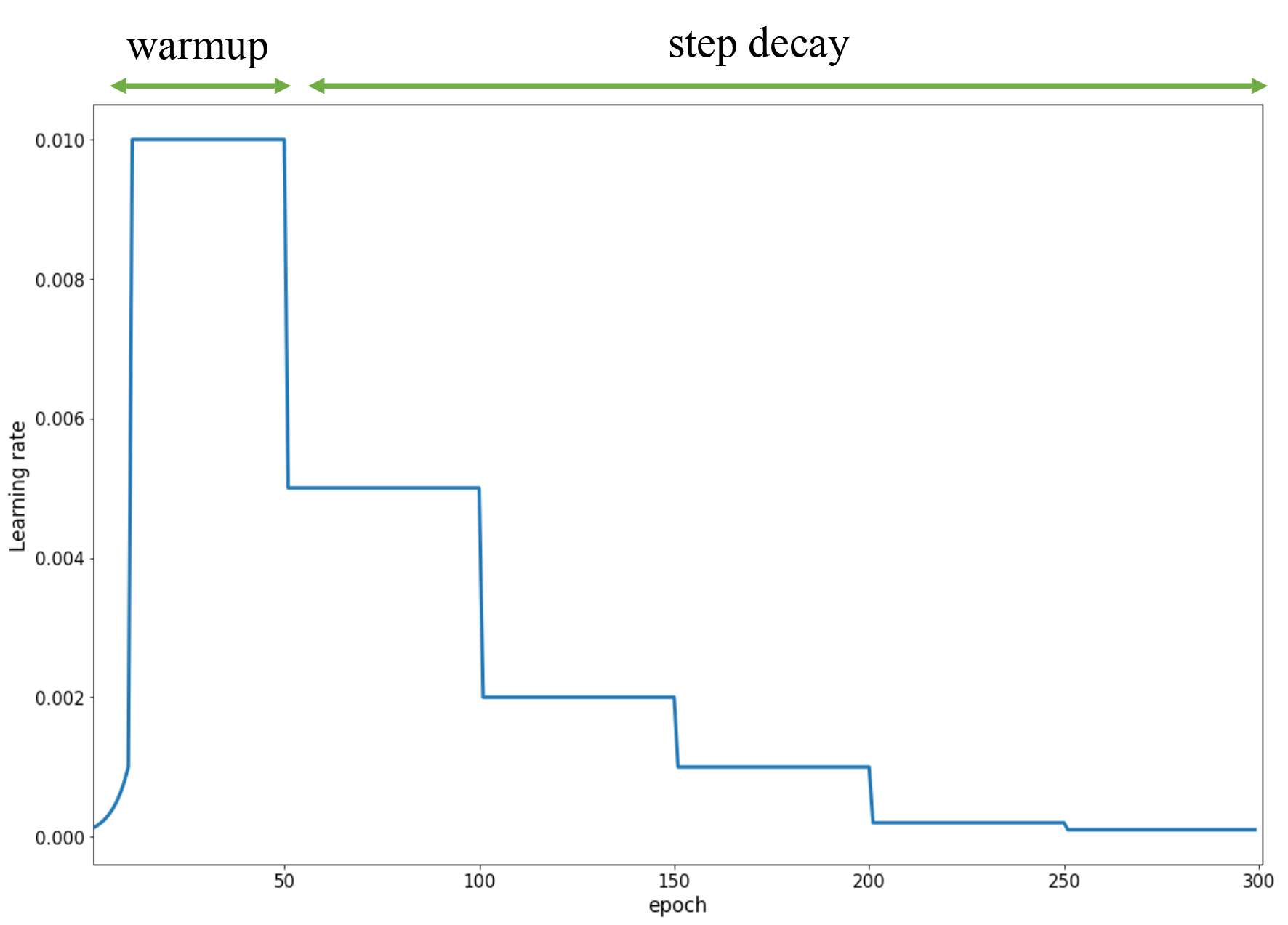
***
